# The Combined Use of Imaging Approaches to Assess Drug Release from Multicomponent Solid Dispersions

**DOI:** 10.1007/s11095-016-2018-x

**Published:** 2016-08-29

**Authors:** Kateřina Punčochová, Andrew V. Ewing, Michaela Gajdošová, Tomáš Pekárek, Josef Beránek, Sergei G. Kazarian, František Štěpánek

**Affiliations:** 1 Department of Chemical Engineering, University of Chemistry and Technology Prague, Prague 6, Czech Republic; 2 Department of Chemical Engineering, Imperial College London, South Kensington Campus, London, SW7 2AZ UK; 3 Zentiva, k.s, U Kabelovny 130, Prague 10, Czech Republic

**Keywords:** amorphous solid dispersion, confocal Raman spectroscopy, crystallisation, FT-IR spectroscopic imaging, magnetic resonance imaging

## Abstract

**Purpose:**

Imaging methods were used as tools to provide an understanding of phenomena that occur during dissolution experiments, and ultimately to select the best ratio of two polymers in a matrix in terms of enhancement of the dissolution rate and prevention of crystallization during dissolution.

**Methods:**

Magnetic resonance imaging, ATR-FTIR spectroscopic imaging and Raman mapping have been used to study the release mechanism of a poorly water soluble drug, aprepitant, from multicomponent amorphous solid dispersions. Solid dispersions were prepared based on the combination of two selected polymers - Soluplus, as a solubilizer, and PVP, as a dissolution enhancer. Formulations were prepared in a ratio of Soluplus:PVP 1:10, 1:5, 1:3, and 1:1, in order to obtain favorable properties of the polymer carrier.

**Results:**

The crystallization of aprepitant during dissolution has occurred to a varying degree in the polymer ratios 1:10, 1:5, and 1:3, but the increasing presence of Soluplus in the formulation delayed the onset of crystallization. The Soluplus:PVP 1:1 solid dispersion proved to be the best matrix studied, combining the abilities of both polymers in a synergistic manner.

**Conclusions:**

Aprepitant dissolution rate has been significantly enhanced. This study highlights the benefits of combining imaging methods in order to understand the release process.

**Electronic supplementary material:**

The online version of this article (doi:10.1007/s11095-016-2018-x) contains supplementary material, which is available to authorized users.

## Introduction

Amorphous solid dispersions are widely used to enhance dissolution rates and absorptions of orally administered formulations of poorly water-soluble drugs. The formation of an amorphous solid dispersion involves the combination of two or more chemically distinct components – typically a poorly soluble, hydrophobic drug and a readily soluble, hydrophilic polymer – into a single matrix (1–3). Amorphous solid dispersions can be formed either by mixing the components in the molten state, followed by cooling (e.g. hot-melt extrusion process), or by dissolving them in a common solvent, followed by rapid evaporation (e.g. spray drying process). Depending upon the nature of the components and their ratio in the matrix, pharmaceutical formulations based on amorphous solid dispersions can suffer from thermodynamic instability, resulting in unexpected crystallization of the drug in the solid state during storage or during dissolution. Consequently, this causes a reduction in the amount of the drug bioavailability (4,5).

The dissolution of tablets formed from amorphous solid dispersions is a relatively complex process where several phenomena occurr simultaneously (6). These include the hydration, water ingress, swelling and erosion of the polymer matrix, as well as the diffusion of the drug across the swollen gel layer and into the bulk solution. As the local drug:polymer:solvent ratio varies in different regions of the dissolving tablet, due to different diffusion coefficients of each component, the drug can reach a locally supersaturated state that leads to crystallization. The crystallization (nucleation and crystal growth) is influenced by multiple factors such as the degree of supersaturation, the viscosity of the polymer gel, and the interfacial energy between the crystal nuclei and the solvent (7). In this context, the polymer plays an important role as it can keep the drug in the supersaturated state and therefore inhibit or delay crystallization (5) through a combination of viscosity and surface-energy effects.

From a design of formulation perspective, the selection of the most suitable polymers must reflect processability during the preparation of amorphous solid dispersions, stability of the amorphous form of drug during storage, and the ability to control drug release and inhibit crystallization during dissolution (8–10). Often, a single polymer will not guarantee all the above-mentioned properties simultaneously. For example, although polymers with a strong affinity towards the drug molecules via hydrogen bonding or hydrophobic interactions could be effective at preventing crystallization (11–16), they might at the same time restrict the ingress of water into the tablet, resulting in sub-optimal release profiles.

In our recent work (9,17,18), we have shown that both Soluplus (an amphiphilic polymer) and polyvinylpyrrolidone (PVP) were able to form stable amorphous solid dispersions with aprepitant at a drug:polymer ratio of 1:3 by weight. However, neither polymer alone could provide an ideal drug release profile. While Soluplus was able to suppress crystallization, the release rate was limited by the slow diffusion of water into the matrix. On the other hand, the release of aprepitant from a PVP matrix was way too fast, resulting in crystallization of the drug. Therefore, it was suggested that a combination of polymers with different (even opposite) properties in a mixed matrix could result in the favorable characteristics from each of the components in the final formulation (19,20).

In order to rationalize the selection of polymers for the mixed-matrix formulations, it is important to understand the underlying mechanism of drug release and the molecular interactions between individual components during dissolution. To this end, it is beneficial to combine standard USP-type dissolution tests with chemically specific, spectroscopic imaging-based analytical approaches such as attenuated total reflection-Fourier transform infrared (ATR-FTIR) spectroscopic imaging (21–24), magnetic resonance imaging (MRI) (25,26), UV imaging (27,28) and Raman imaging (29,30). These techniques allow the visualization of dynamic physico-chemical processes within a tablet under dissolution conditions, making it possible to elucidate phenomena that could not be easily identifiable from the USP release curve. Since each imaging method is based on different physical principles with a corresponding difference in the chemical, spatial and temporal resolution, their combination may be necessary to reveal a full picture of the dissolution process (17,31,32).

**Fig. 1:**
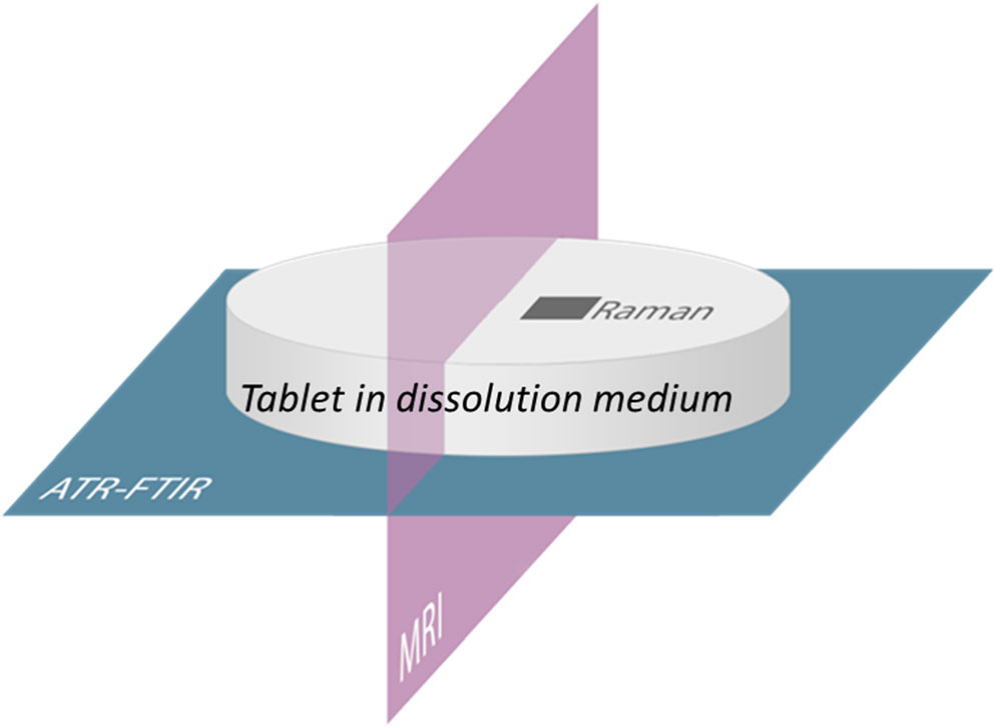
Scheme of image positions relative to the tablet, provided by each imaging method used in this work.

The aim of the present work is to demonstrate – for the first time – the combination of three chemical imaging methods (MRI, ATR-FTIR spectroscopic imaging, and confocal Raman mapping) in order to understand the behaviour of drug release from amorphous solid dispersion in a mixed polymer matrix. Each imaging method provides a different view (in terms of spatial information and chemical specificity) of the dissolving tablet (Fig. 1). Using specific model formulations containing aprepitant as the drug with Soluplus and PVP as polymers, we show that a wealth of information can be gained about the dissolving tablets using a combination of these approaches to reveal insight about drug relase that none of the methods can provide individually. We can design a formulation such that the drug relase rate from the mixed polymer matrix is faster than from either of the polymers alone, and at the same time the crystallisation of the drug is suppressed.

## Materials and Methods

### Materials

The drug aprepitant was kindly provided by Zentiva, k.s. (Prague, Czech Republic). Aprepitant is a poorly water-soluble drug (category II) according to the Biopharmaceutics Classification System (BCS) criteria. Two different polymers were used as matrix materials for the amorphous solid dispersions. Polyvinylpyrrolidone K30 (PVP), obtained from BASF (Germany), is a water soluble polymer with a molecular weight of 30 000 g/mol. Soluplus (polyvinyl caprolactam–polyvinyl acetate–polyethylene glycol graft copolymer), obtained from BASF (Germany), is an amphiphilic, solubility enhancing excipient with an average molecular weight of 118 000 g/mol.

### Preparation of Solid Dispersions

Amorphous solid dispersions were prepared by spray drying. The drug:polymer ratio in the solid dispersions was fixed at 1:3 by weight, where the polymer matrix was composed of systematically varying Soluplus:PVP ratios ranging from 1:0, 1:1, 1:3, 1:5, 1:10 to 0:1. To prepare the amorphous dispersion, aprepitant (1.0 g) was dissolved in ethanol (150 ml), the solution was mixed at 40°C for 15 min, and the required amount of the polymers was added until complete dissolution to achieve an overall drug:polymer ratio of 1:3 *w/w*. The solution was spray dried using the Mini Spray Dryer B-290 (Büchi, Switzerland) with an inert nitrogen loop. The spray-dried particles were subsequently compressed to tablets (140 mg, round flat shape, 7 mm in diameter) at a compression force 5 kN. In addition to a formulation where both polymers and the drug were spray dried together, tablets compressed from a physical blend of spray dried amorphous solid dispersion made of aprepitant:PVP in ratio 1:3, and admixed spray dried particles of pure Soluplus, were formed.

### Differential Scanning Calorimetry

The glass transition temperatures of the solid dispersions were measured by modulated temperature differential scanning calorimetry (MTDSC) immediately after preparation. DSC measurements were performed on a TA Instruments, Discovery DSC apparatus. The samples were weighed in aluminum pans (40 μl), covered and measured in a nitrogen flow. Investigations were performed in a temperature range of 0 to 300°C with a heating rate of 5°C/min (amplitude = 0.8°C, period = 60 s). The average weight of the sample was approximately 4–5 mg.

### Magnetic Resonance Imaging

The Magnetic Resonance Imaging (MRI) Desktop System Icon (Bruker BioSpin, Germany) was used to observe the water ingress into tablets and structural changes in the gel layer during dissolution. The MRI analysis was based on multi-slice-multi-echo (MSME) sequences with echo time 25 ms, repetition time 1500 ms, number of averages 2, number of repetitions 1. The images were weighted by relaxation times T1. The resolution of the images was 128 × 128 pixels for a field of view 1.8 × 1.8 cm. The slice thickness was 1 mm. The first scan was used to localize the position of the tablet in the flow cell and choose the number, position and thickness of slices. The dissolution medium was water at a flow rate of 5 ml/min and room temperature. The scans were taken every 8 min. Further details of the experimental set-up can be found in (33).

### Attenuated Total Reflection Fourier Transform Infrared (ATR-FTIR) Spectroscopy and Spectroscopic Imaging

FTIR spectra for all of the pure material and formulations studied in this investigation were measured using an Alpha-P spectrometer (Bruker, UK) in ATR mode, fitted with a diamond crystal. Spectra were recorded across the range of 4000–600 cm^−1^, using a spectral resolution of 8 cm^−1^ and 32 co-added scans.

To collect ATR-FTIR spectroscopic images of the dissolving tablet compacts, an ATR accessory (Pike, USA) fitted with a zinc selenide (ZnSe) crystal was employed. This ATR accessory was placed in an IMAC sampling compartment that was attached to an FTIR spectrometer (Equinox 55, Bruker) and the imaging data was recorded using a focal plane array (FPA) detector. Spectra in the mid-IR region between 4000 and 900 cm^−1^ was recorded for all of the dissolution experiments using a spectral resolution of 8 cm^−1^ and 32 co-added scans. The FPA detector was setup to record an array size of 96 × 96 pixels, meaning that 9216 individual FTIR spectra were recorded in a single experiment. This resulted in spectroscopic images with dimensions of approximately 7.75 × 6.05 mm^2^ and a spatial resolution of 100–150 μm (34).

The measuring surface of the ZnSe crystal has a diameter of 20 mm which allowed one to obtain information from the entire 3 mm tablet as well as the surrounding solution. Spectroscopic images representing the spatial distribution of the different components during the experiment were generated based on the identification of specific absorption bands for each of the materials of interest. Subsequently, the integrated absorbance of these spectral bands was plotted as a function of the measured area. Table I shows the integration ranges of spectral bands used to generate the spectroscopic images of the different components of interest in this investigation. The spectral band integration method used draws a linear baseline between the frequency limits defined in Table I and the area above this line is integrated. It should be realised that red regions in the spectroscopic images relate to high absorbance and the blue areas relate to low absorbance of the species.

**Table I:** The Specific Integration Ranges of Spectral Bands Used to Generate ATR-FTIR Spectroscopic Images for the Different Components of Interest in this Investigation

Components	Integration range/cm^−1^	Spectral band peak/cm^−1^
Crystalline aprepitant	985–1010	998
Soluplus	1215–1250	1224
PVP	1305–1330	1313
Water	3000–3600	3300

### Dissolution Methodology Under ATR-FTIR Spectroscopic Imaging

The dissolution experiments were setup by positioning the tablet compact in the centre of the 20 mm ZnSe crystal. A custom designed Perspex flow cell (35) placed above the tablet and a rubber O-ring was used to form a seal between the ZnSe crystal and the flow cell. Furthermore, the Perspex flow cell was used to provide sufficient pressure from the top of the tablet tablet (3 mm in diameter) that allowed contact to be achieved that allowed contact to be achieved between the sample and the ATR crystal. Good contact is needed for collection of reliable spectral information when using the ATR sampling methodology since the penetration of the evanescent IR beam is typically up to 10 μm beyond the surface of the crystal.

The dissolution medium (distilled water) was pumped through the flow cell at a rate of 5 ml/min. It should be realised that when using this setup the tablet compact is sandwiched between the flow cell and the measuring surface of the ZnSe crystal, meaning that the dissolution medium only contacts the side, not the top or bottom, surface of the tablet.

### Raman Spectroscopy

The spectra of pure components were measured by the inVia Reflex confocal Raman microscope (Renishaw, UK) at an excitation wavelength of 785 nm (operated at laser power of 50 mW) with an integration time of 0.5 s. The spectral range was 1800–730 cm^−1^ and the spectra were obtained from amorphous spray dried powder or the initial crystalline drug.

### Raman Mapping

*In situ* dissolution behavior of amorphous solid dispersions was studied using the inVia Reflex confocal Raman microscope (Renishaw, UK) with a specifically designed cells enabling the measurement of dissolution under water in stagnant conditions or under flow (for details of the flow cell, see Supplementary Information 1). The dissolution medium was distilled water at room temperature and a flow rate of 5 ml/min. The x-y surface area scans were used to measure the possible crystallization of aprepitant during dissolution every 5 min. By focusing on the surface of the tablet at each time interval (the tablet swells or dissolves upon dissolution, therefore the z-coordinate of the imaging area must be adjusted), x-y surface area scans (20 × 20 μm^2^), with a 2 μm step size using a 48× immersion objective were performed. Subsequently, the spectral data sets were background and cosmic rays corrected, processed by the intensity of unique bands of amorphous (1007 cm^−1^) and crystalline (1043 cm^−1^) aprepitant, and converted to false-color images by the software Wire 4.1.

### USP Dissolution Testing

In addition to imaging-based dissolution experiments (MRI, ATR-FTIR spectroscopic imaging, Raman mapping), standard *in vitro* dissolution testing was performed according to the United States Pharmacopeia (USP) type I method. The dissolution tests were conducted using a Sotax semi-automated system (Sotax AT7) with a validated analytical method (HPLC Waters 2695 Alliance with UV detection). The samples were filtered using a single filter with a pore size of 40 μm. The dissolution profile was measured with baskets at 100 rpm in 150 ml of distilled water at room temperature. The concentration of aprepitant in the solution was determined at sampling intervals ranging from 30 to 120 min for a period of up to 510 min.

## Results and Discussion

### Solid State Characterization of the Solid Dispersions

The amorphous nature of the formulations prepared as described in section 2.2, where the drug is molecularly dispersed in the polymer matrix, was confirmed by DSC analysis. The glass transition temperatures of the spray-dried materials with varying composition of the polymer matrix were 138.0, 135.4, 134.0, and 127.9°C for a Soluplus:PVP ratio of 1:10, 1:5, 1:3, and 1:1, respectively. Usually, the Tg curve obeys the Gordon–Taylor equation. Tg decreases from the pure PVP with Tg 159.5°C to pure polymer Soluplus (Tg 68.2°C) depending on the ratio of both polymers in carrier. Tg of amorphous pure Aprepitant is 93.3°C. It should be realized that the aprepitant:polymer ratio was kept constant in all cases, i.e. the above ratios refer to the composition of the polymer matrix only, which represents 75% of the formulation on a mass basis. As reported in our previous work (18), the glass transition temperatures of solid dispersions with pure polymers were 142.9 and 57.8°C in the case of aprepitant:PVP and aprepitant:Soluplus, respectively.

### Magnetic Resonance Imaging (MRI)

**Fig. 2:**
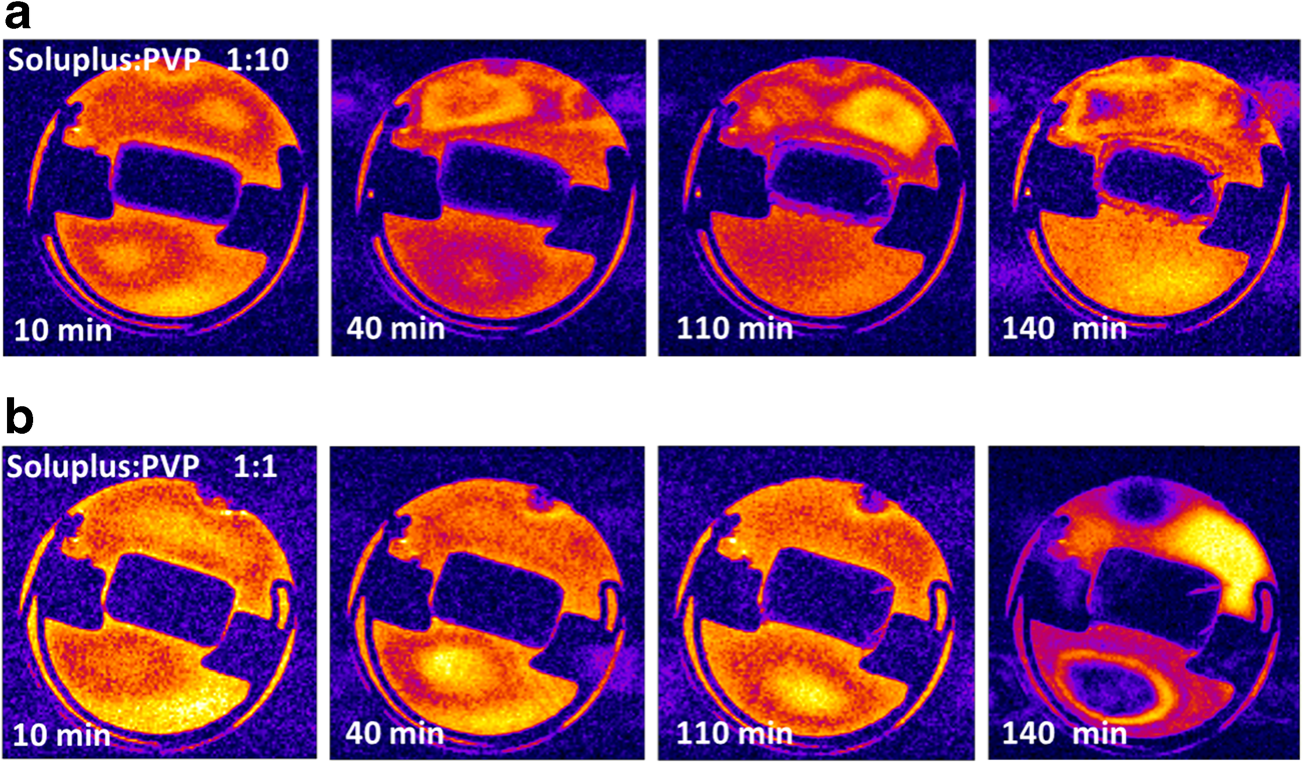
Images of whole-tablet dissolution obtained by MRI. The composition of the polymer matrix was (**a**) Soluplus:PVP 1:10, and (**b**) Soluplus:PVP 1:1.

**Fig. 3:**
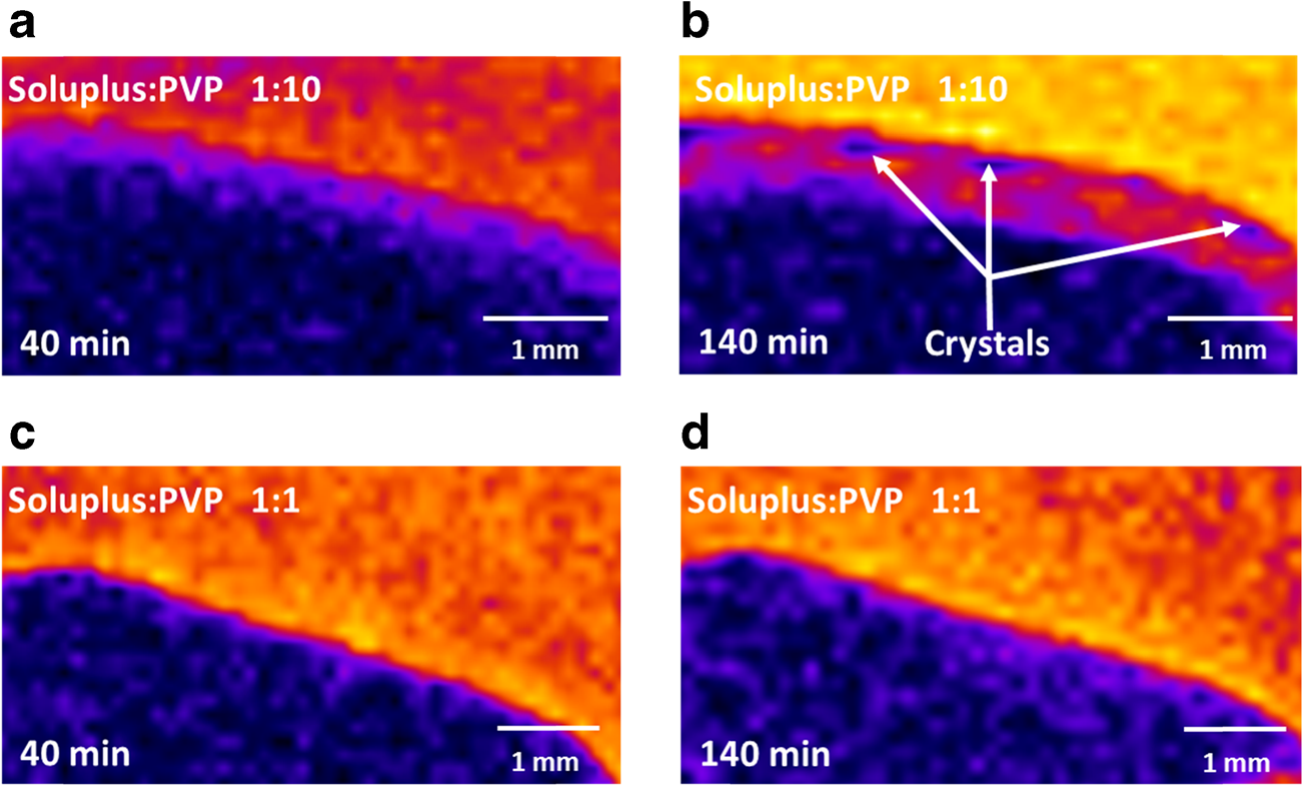
Detail of the interface between the tablet surface during the dissolution experiments obtained by MRI, for polymer matrix compositions and times as indicated in the panels.

MRI analysis enables the observation of phenomena at the length-scale of the whole tablet in the 3D space. Hence, the behavior of the tablet is not restricted by the close physical proximity of the experimental setup such as the contact with an ATR crystal when performing ATR-FTIR spectroscopic imaging studies. Specifically, MRI enables the observation of water ingress into the tablet, polymer swelling and erosion, as well as potential structural changes in the hydrated polymer layer. The evolution of the tablet structure in two cases (Soluplus:PVP ratio 1:10 and 1:1), visualized by MRI, is summarized in Fig. 2. The combination Soluplus:PVP 1:10 (Fig. 2a) shows a recognizable dry core and the formation of a gel layer of increasing thickness during dissolution. The gel layer contains a higher concentration of hydrogen atoms associated with water, corresponding to longer relaxation times. In contrast, the dry core of the tablet is characterized by very short relaxation times, indicated by dark (black) color in the color scale used in Fig. 2. Details of the gel layer are shown in Fig. 3a and b. Interestingly, the detailed view (Fig 3b) reveals the occurrence of small regions with shorter relaxation times that can be clearly detected after 110 min of dissolution in the gel layer. It can be hypothesized that these regions correspond to a newly formed solid phase. During the dissolution of amorphous solid dispersions, the drug can become locally supersaturated in the gel layer and crystallize. Since magnetic resonance imaging is not a suitable method to identify chemically specific information about the particles, the exact nature of the particles has to be identified by other imaging methods.

In contrast, the polymer carrier with a Soluplus:PVP ratio 1:1 shows a substantially lower rate of tablet hydration (Fig. 2b). No gel layer is formed around the tablet after contact with water within the 140 min time frame of the experiment (Fig. 3d). It seems that water penetrates to the tablet preferentially through cracks formed during dissolution (upper right-hand corner of the tablet in Fig. 2b). The lower hydration rate of the tablet can be attributed to lower hygroscopicity of Soluplus, which represents a higer proportion of the polymer matrix in this case. Crucially, MRI did not indicate any crystallization processes during the dissolution of tablets made with a Soluplus:PVP ratio 1:1 (Fig. 3d).

### ATR-FTIR Spectroscopic Imaging

ATR-FTIR spectroscopic imaging has a significant advantage of providing chemically specific information about the solid dispersion components. It simultaneously measures thousands of infrared spectra and provides spatially resolved quantitative information about the concentration of the individual components in the measured area. A potential disadvantage of ATR-FTIR spectroscopic imaging is the need to physically press the tablet against the ATR crystal, which may influence the natural dissolution mechanisms by constraining water ingress and gel layer formation to just the outside surface of the tablet.

**Fig. 4:**
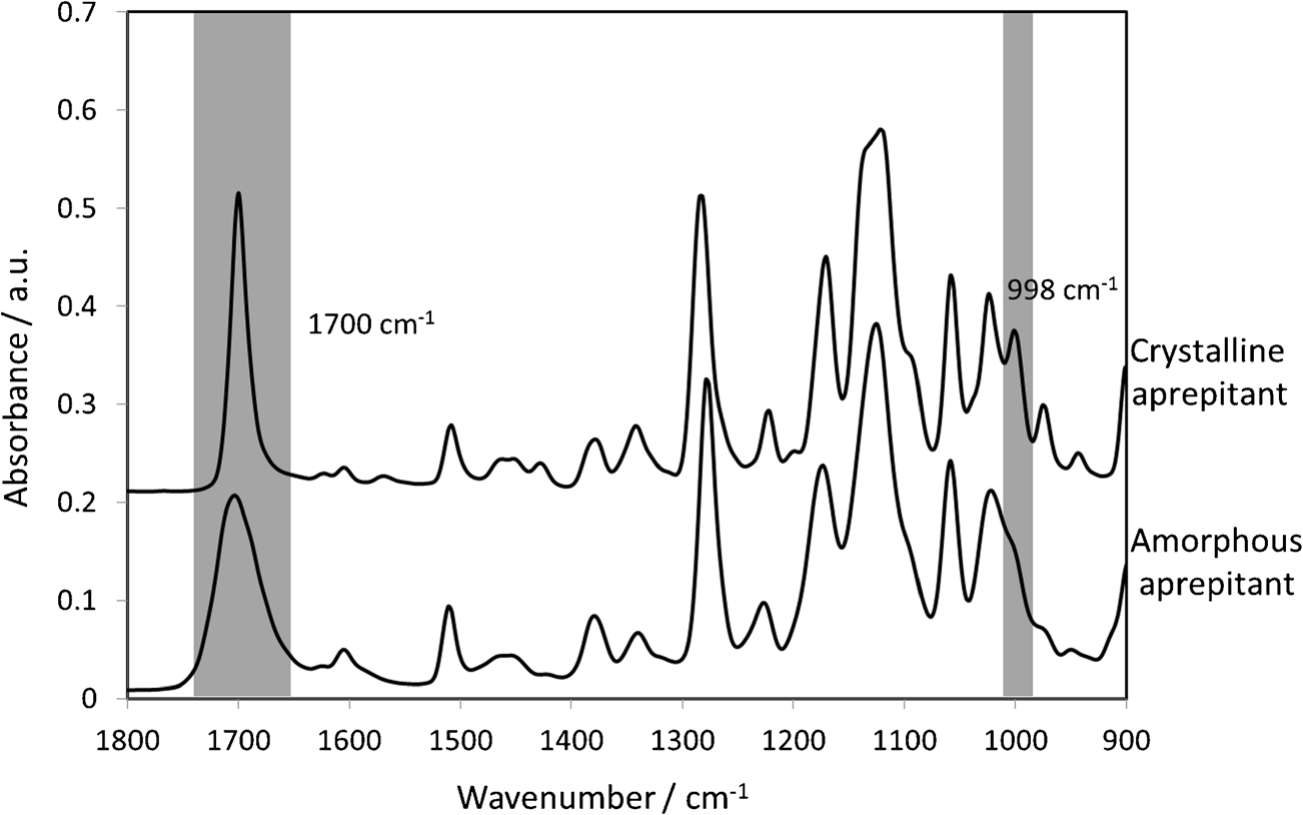
ATR-FTIR spectra recorded from measurement of pure crystalline and amorphous aprepitant. Two significant spectral changes between the structural forms, the appearance of the band at 998 cm^−1^ and the narrowing of the band at 1700 cm^−1^, are highlighted.

The structural change of a drug from an amorphous state to its crystalline form can influence its infrared spectrum. The comparison of ATR-FTIR spectra of the amorphous and crystalline form of aprepitant is shown in Fig. 4. Two significant spectral changes between the structural forms are the appearance of the band at 998 cm^−1^ and the narrowing of the band at 1700 cm^−1^. These differences in the spectrum can be used to characterize and detect the crystallization from the amorphous solid dispersion. Thus, it is feasible that this approach can complement the initial observations of the MRI by confirming the presence of any structural changes during the tablet dissolution experiment. ATR-FTIR spectroscopic imaging also provides insight into possible causes of crystallization. Local supersaturation of the drug, which is a pre-requisite of crystallization, can be caused by the local depletion of a polymer that originally stabilised the amorphous form (18). Due to the high chemical specificity of ATR-FTIR spectroscopic imaging is it possible to reveal changes in the local polymer concentrations during dissolution by selection of unique absorption bands for these components.

**Fig. 5:**
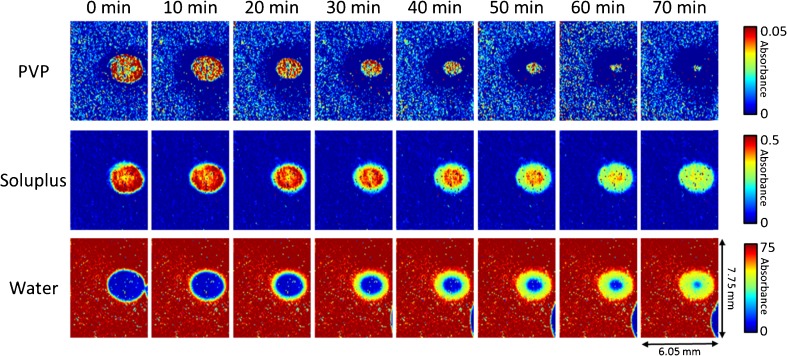
ATR-FTIR spectroscopic images of tablet compact from amorphous dispersion (ratio 1:5) during dissolution. Images representing the spatial distribution of the two polymers used in the formulations, PVP (*top row*) and Soluplus (*middle row*), and the penetration of water (bottom row) into the tablet compact. The dimensions of the images are approximately 7.75 × 6.05 mm^2^.

ATR-FTIR spectroscopic images representing the spatial distribution of the two polymers used in the amorphous solid dispersion with carrier made of Soluplus:PVP 1:5, and the water penetration into the tablet compact during dissolution are shown in Fig. 5. PVP (top row) is dissolved rapidly from the tablet immediately after contact with water. After 70 min of dissolution, there is almost no PVP remaining in the tablet and it is almost entirely wet. The penetration of water (bottom row) into the tablet compact is coincident with the loss of PVP. Thus, it is evident that PVP readily dissolves in spite of the presence of other components in the amorphous solid dispersion. The high amount of PVP in the solid dispersion significantly improves the water penetration rate but at the same time, its rapid depletion can result in a high local supersaturation of the drug. Soluplus (middle row) forms a gel layer after water penetration into the tablet. In contrast to PVP, only a slow depletion of Soluplus concentration was observed in 70 min.

It can be expected that the depletion rate of PVP from the mixed polymer matrix would depend on the ratio of the two polymers. The depletion process depends on the rate of hydration, polymer chain disentanglement and diffusion of each polymer through the composite matrix.

**Fig. 6:**
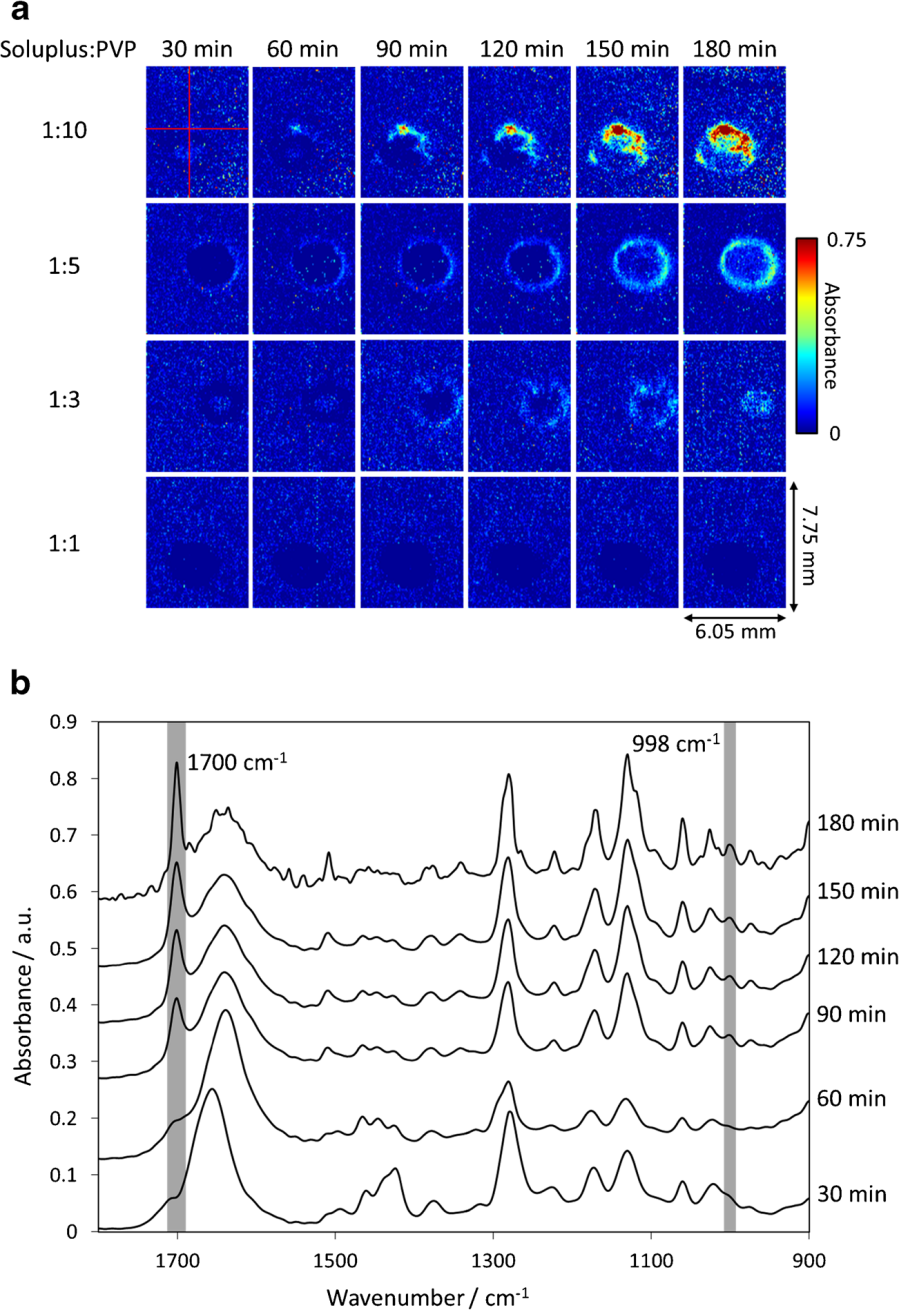
(**a**) ATR-FTIR spectroscopic images showing the presence of crystalline aprepitant that appeared during the dissolution of the tablet compacts prepared using formulations with a varying Soluplus:PVP ratio as indicated. The dimensions of the images are approximately 7.75 × 6.05 mm^2^. (**b**) ATR-FTIR spectra extracted from the same location during dissolution of the carrier with Soluplus:PVP 1:10. The specific location is indicated by the crosshair in case a). The appearance of spectral bands indicative of the formation of crystalline aprepitant, at 998 and 1770 cm^−1^, as the experiment progressed, are highlighted.

In order to establish the extent to which the dissolution and depletion of PVP from the tablet matrix leads to crystallization of the drug, ATR-FTIR spectroscopic images based on the characteristic spectral bands of the crystalline form of aprepitant were compared for Soluplus:PVP ratios ranging from 1:1 to 1:10. The ATR-FTIR spectroscopic images (Fig. 6a) reveal the occurrence of crystalline aprepitant in all formulations except that with a Soluplus:PVP ratio 1:1 (bottom row in Fig. 6a) where no crystalline phase was detected even after 180 min of dissolution. In those cases where aprepitant crystallization did occur, the quantity of the crystalline phase and its spatial distribution varied as function of the Soluplus:PVP ratio in the polymer matrix. For the lowest Soluplus:PVP ratio 1:10, a single nucleation event seems to have occurred at a point indicated by the crosshair (Fig. 6a, top row, t = 30 min), from which a growing cluster of the crystalline phase agglomerates. The ATR-FTIR spectra extracted from the spectroscopic images recorded from that point at different times are depicted in Fig. 6b. The spectral changes, in particular the appearance of unique bands at 998 and 1770 cm^−1^, indicate the local crystallization of aprepitant in the region of interest. The formation of a distinct crystalline phase shown in Fig. 6a and confirmed in the extracted spectra in Fig. 6b can be regarded as a proof that the solid particles apparent in the MRI sequence (Figs. 2a and 3b) were indeed aprepitant crystals.

For the Soluplus:PVP ratio 1:5, the crystalline phase occurs more symmetrically in a circular region corresponding to the periphery of the original tablet (Fig. 6a). The intensity is lower (despite identical drug load in the tablet), meaning that crystallisation was partially suppressed. Increasing the Soluplus:PVP ratio to 1:3 results in a further delay of the onset of crystallization, to a point that the crystalline phase no longer forms a hollow circular ring, but is restricted to a central area of the original tablet. Finally, the highest Soluplus:PVP ratio 1:1 was able to suppress crystal formation altogether beyond 180 min, which is again complementary to the observations made using MRI (Figs. 2b and 3d).

The formation of solid particles in the gel layer was observed by MRI in the previous section and identified by ATR-FTIR spectroscopic imaging as the crystalline form of aprepitant. However, the spatial localization of the crystallization event in the case of the Soluplus:PVP ratio 1:10 is indicative of a possible heterogeneous nucleation, which could be influenced by the physical presence of the ATR crystal. Heterogeneous nucleation typically occurs at lower supersaturation levels than homogeneous nucleation. Therefore, the crystallization of aprepitant could be initiated and observed sooner in the ATR-FTIR spectroscopic images because of the experimental setup in which the tablet is in direct contact with the ATR crystal that may provide nucleation points. This is in contrast to a tablet in an unrestricted environment such as the MRI dissolution cell (cf. Fig. 2). Furthermore, the spatial resolution of ATR-FTIR images is 100–150 μm for the specific optical configuration used in this study, so any initial crystallization sites forming that are significantly smaller than this size may not be resolved.

### Raman Microscopy

The experimental setup of the dissolution cell in Section 2.8 makes it possible to observe any structural changes (particularly drug crystallization) at the free tablet surface in contact with the dissolution medium. The surface of the tablet is not limited for water penetration or dissolution of drug, and there is no foreign surface for preferred heterogeneous nucleation. Differentiation of the amorphous and crystalline form of drug was achieved based on their individual Raman spectra. Unique bands used for the differentiation of the amorphous drug, crystalline drug, Soluplus and PVP were 1005, 1047, 1450 and 935 cm^−1^, respectively, similarly to those described in (18).

**Fig. 7:**
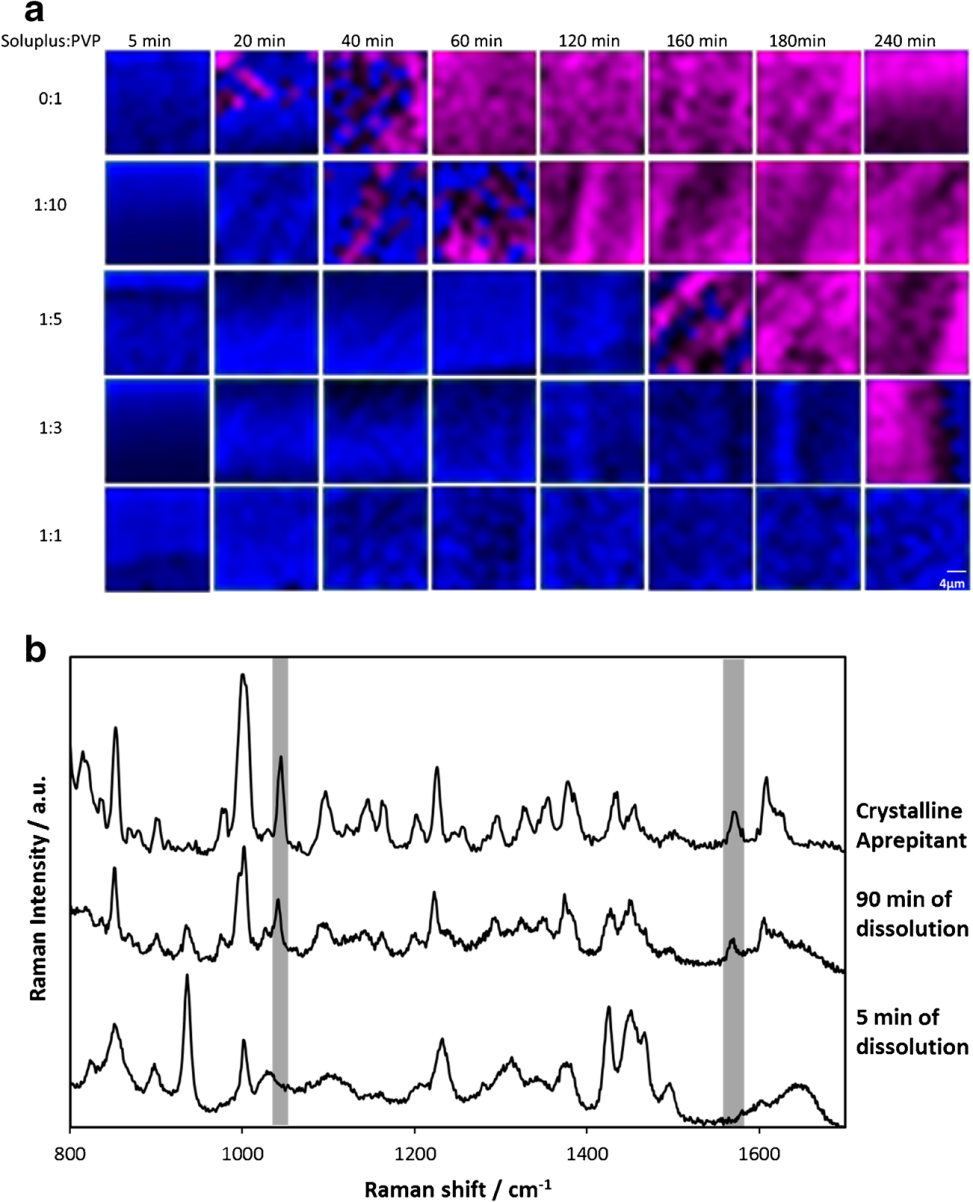
(**a**) Raman imaging of how the distribution of the crystalline aprepitant changes during dissolution (x-y surface area scans covering 20 × 20 μm^2^). The false-color images depict the solid dispersions of each combination in dark blue, and pure crystalline drug in pink, respectively. (**b**) Raman spectra extracted from the same location of the images during dissolution of tablets with a Soluplus:PVP ratio 1:10. The appearance of spectral bands indicative of the formation of crystalline aprepitant, at 1047 and 1574 cm^−1^, as the experiment progressed are highlighted.

False-color images based on the Raman spectra obtanied from x-y scans of the tablet surface are shown in Fig. 7a. Crystallization was identified after 20 min for the aprepitant:PVP matrix (i.e. when no Soluplus is added to the formulation). The increased weight loading of Soluplus in the carrier delays the onset of crystallization of aprepitant to 40, 160, and 240 min for the Soluplus:PVP ratio of 1:10, 1:5, and 1:3 solid dispersions, respectively. Crystallization of aprepitant was not observed in the polymer matrix based on Soluplus:PVP ratio of 1:1 (bottom row in Fig. 7a) throughout 240 min of dissolution. The appearance of spectral bands at 1047 and 1574 cm^−1^, which is indicative of formation of crystalline aprepitant during dissolution, is represented in Fig. 7b.

**Fig. 8:**
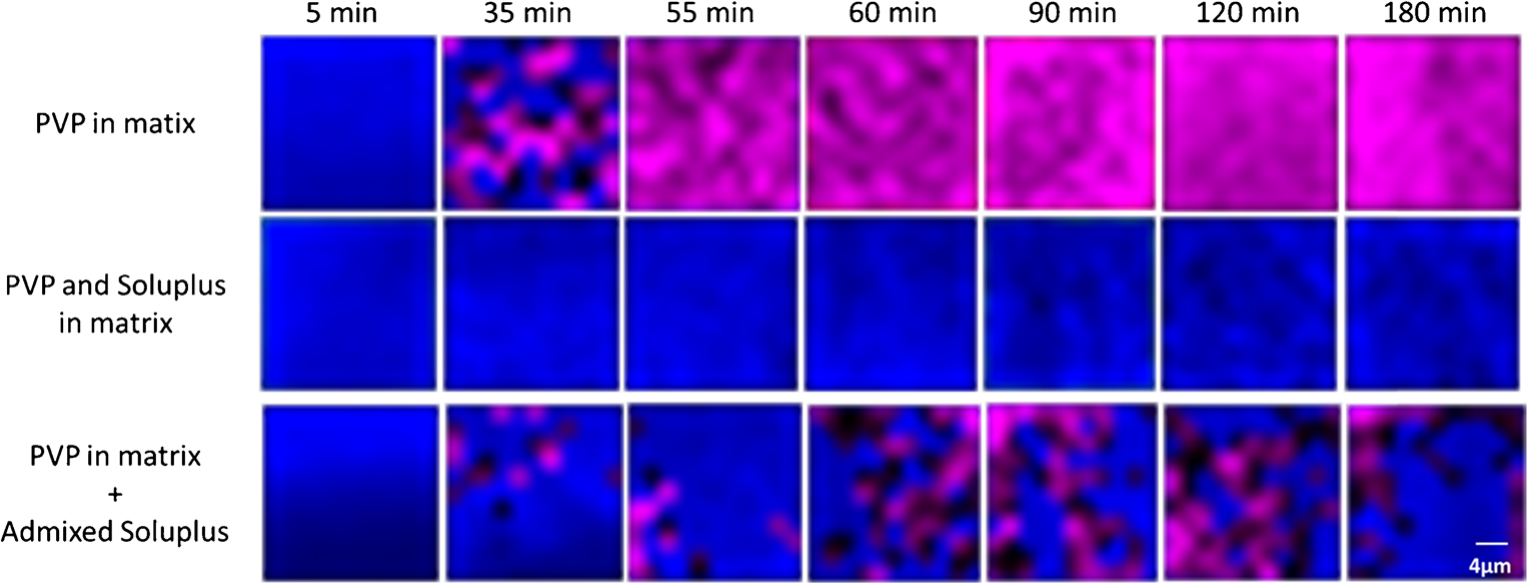
Raman images of a tablet surface at different times during dissolution showing the effect of Soluplus on the inhibition of crystallization. False-colors indicate crystalline drug in pink and amorphous solid dispersion in blue. The top row is a solid dispersion of aprepitant:PVP 1:3 in the spray-dried matrix without Soluplus, the bottom row represents the same spray-dried dispersion (aprepitant:PVP 1:3) but with physically admixed Soluplus (the final ratio of polymers in the tablet is 1:3 Soluplus:PVP), and the middle row shows the aprepitant solid dispersion with a Soluplus:PVP 1:3 combined directly in the spray-dried dispersion (i.e., nothing added externally). The dimensions of each image are 20 × 20 μm^2^.

The results from the Raman mapping follow those from the other spectroscopic imaging approaches, that Soluplus is responsible for the suppression or crystallization in the mixed polymer matrix. The question is whether the effect of Soluplus on the inhibition of crystallization depends on the manner in which Soluplus is incorporated into the tablet matrix. To find out, we have compared formulations without Soluplus (aprepitant:PVP 1:3), with Soluplus directly incorporated into the spray-dried solution, and a tablet matrix composed of admixed spray dried particles of pure Soluplus with solid dispersion particles containing aprepitant:PVP 1:3. The examples of false-color Raman images (Fig. 8) show that the amorphous solid dispersion without Soluplus had significantly crystallized by 35 min (top row). On the other hand, the presence of Soluplus in the carrier (bottom row) can inhibit the crystallization of aprepitant. Interestingly, admixed Soluplus (middle row), which is not present directly in the amorphous solid dispersion of the drug, is also able to suppress crystallization in local regions for 3 h. However, this suppression is not uniform as in the case of co-spray dried Soluplus and PVP, but rather only in specific regions where other areas show the crystallization of aprepitant after 35 min, which is a comparable timeframe to the crystallization in aprepitant:PVP 1:3 without Soluplus.

From the formulation perspective, the inhibition of aprepitant crystallization is possible even with admixed Soluplus. However, the amount of admixed Soluplus in the final formulation would have to be significantly higher than the amount of Soluplus in the solid dispersion carrier in order to achieve comparable inhibition of crystallization (for details of the effect of admixed Soluplus, see Supplementary Information 2).

### USP Dissolution Testing

**Fig. 9:**
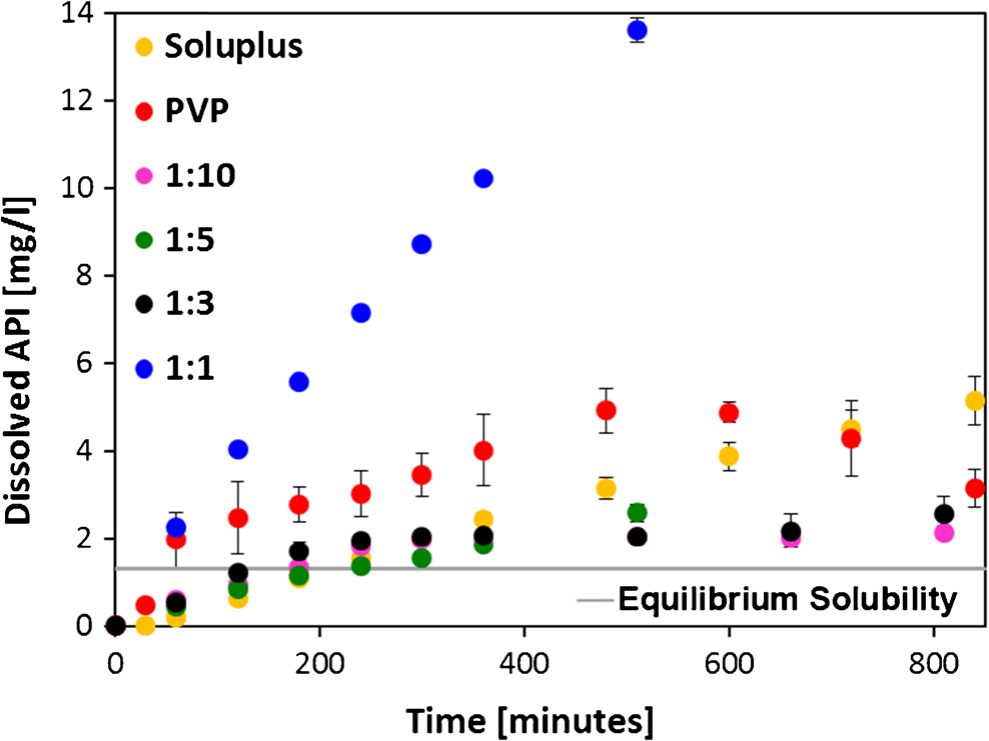
USP dissolution profiles of aprepitant from tablets compressed of spray-dried particles of amorphous solid dispersions of aprepitant in Soluplus and PVP and their mixtures in matrix.

The USP dissolution profiles of aprepitant from the amorphous solid dispersions in pure polymers and their combinations are shown in Fig. 9. The dissolution profile from the pure Soluplus matrix (yellow points) is rather slow, but controlled. Aprepitant dissolution from a pure PVP matrix (red points) was very fast during the first 60 min but then slowed down, to a similar dissolution rate as the pure Soluplus matrix until 480 min. After approx. 480 min, aprepitant precipitation was manifested by a decrease in its concentration in the dissolution medium. The equilibrium solubility of aprepitant in both PVP and Soluplus solutions was measured, and found to be below 0.20 mg/l (detection limit of the HPLC method) for PVP (polymer concentration 800 mg/l, which corresponds to a fully dissolved tablet), and 1.31 mg/l for Soluplus (800 mg/l polymer concentration) (18). This means that the solution was supersaturated with respect to aprepitant, as shown in Fig. 9.

The multicomponent carriers of the solid dispersions, namely the ratio 1:10, 1:5, and 1:3, show a slow drug dissolution rate that is very similar to dissolution from the pure Soluplus matrix. However, after approx. 360 min, the dissolution rate of aprepitant was observed to slow down even further. The most ideal dissolution profile for Aprepitant was obtained for the combination Soluplus:PVP 1:1, where the dissolution rate of drug is considerably enhanced. Dissolution rate during the first 60 min is as fast as that from pure PVP, but this trend continues throughout the duration of the experiment and does not slow down even after 480 min. In this combination, the favorable properties of both polymers are manifested. The solubilisation effect of Soluplus inhibits drug precipitation in solution, while PVP improves the dissolution rate by its fast dissolution from the carrier.

## Conclusion

The combination of three spectroscopic imaging methods MRI, ATR-FTIR spectroscopic imaging, and Raman mapping were succesfully employed to understand the mechanism of drug release from multicomponent amorphous solid dispersions. Each approach was found to complement each other and reveal important information about the tablet dissolution process. Specifically, MRI provides information about the rate of dissolution medium penetration into the tablet and the kinetics of swelling and erosion of the gel layer. ATR-FTIR spectroscopic imaging makes it possible to distinguish and characterise the individual components that make up the tablet formulation, including the structural form of the API and individual excipients. It provides information about the evolution of concentration profiles within the tablet and reveals the diffusion rate of the individual components through the tablet matrix. Raman imaging provies information about the local composition and various phase transitions (e.g. crystallization) that may occur on the surface of the tablet in contact with the dissolution medium. Finaly, a USP dissolution test provides standardized quantitative information about the rate of drug release. These techniques together provide an explanation to the phenomenon of drug crystallization during dissolution and show a global picture about the different water penetration and polymer dissolution rates that none of the techniques alone could conclusively determine.

In the specific case of aprepitant release from a mixed-matrix tablet, the Soluplus:PVP ratio 1:1 in the amorphous solid dispersion has been identified by the *in vitro* spectroscopic imaging approaches and dissolution tests as the best matix combining the favorable properties of both polymers for aprepitant dissolution. The drug dissolution rate has been significantly enhanced, and at the same time the drug has not precipitated during dissolution.

Crystallization was succesfully detected by each imaging technique. More specifically, MRI was able to evaluate a newly formed solid phase in gel layer while the spectroscopic imaging methods (ATR-FTIR spectroscopic imaging and Raman mapping) determined the crystallization due to the structural changes of an amorphous to a crystalline state, manifested and characterized in their respective spectra.

Although the present work has dealt with specific compounds (aprepitant as the API and Soluplus:PVP as polymeric excipients), the methodology presented in this work may be generalized for the development and optimization of other API’s and formulations. As a general guideline for the incorporation of imaging methods into formulation development of amorphous solid dispersions with a mixed polymer matrix, it could be recommended to apply the following steps:

(i) Prepare amorphous solid dispersions with pure polymers and their mixtures, characterize their solid state behaviour and stability by standard solid-state characterisation methods (XRD, DSC, DVS). Determine the maximum amount of API in the matrix that still forms stable amorphous solid dispersion.
(ii) Carry out dissolution tests from the amorphous solid dispersions and note any “unusual” behaviour such as a change in rate of the dissolution curve or a decrease of concentration in time, which could signal drug crystallisation or other drug release inhibiting phenomena.
(iii) Use MRI to determine the rate of dissolution medium penetration into the tablet. Using mass balance and the USP release curve, determine if the matrix hydration rate is the rate-limiting step. If so, consider a change of formulation to enhance the rate of penetration.
(iv) Use ATR-FTIR spectroscopic imaging to observe the concentration profiles of the API and individual excipients in the hydrated tablet matrix. Use the ATR-FTIR spectra to identify interactions between the API and excipients that might influence the API diffusion rate and/or its tendency to crystallise.
(v) If there is a suspicion of API crystallisation during dissolution, use Raman mapping with surface x-y scans to identify the presence of the crystalline phase and evaluate the influence of formulation variables on the timing and extent of API crystallisation at the surface of the tablet.

It should be realised that each API and formulation has its specific behaviour and the time pressure of formulation development in the industrial context may not always allow a full and rigorous analysis to be performed. Nevertheless, we hope to have shown that the combination of standard USP dissolution tests with several complementary spectroscopic imaging methods is a powerful approach that can reveal the mechanisms and phenomena that govern drug release from amorphous solid dispersions.

## Electronic supplementary material

Below is the link to the electronic supplementary material.

Supplementary Information 1 (DOCX 396 kb)

Supplementary Information 2 (DOCX 1120 kb)

## Abbreviations

ATR-FTIR: Attenuated total reflection-Fourier transform infrared spectroscopy
BCS: Biopharmaceutics classification system
DSC: Differential scanning calorimetry
DVS: Dynamic vapour sorption
FTIR: Fourier transform infrared spectroscopy
HPLC: High performance liquid chromatography
MRI: Magnetic resonance imaging
MSME: Multi slice multi echo
MTDSC: Modulated temperature differential scanning calorimetry
PVP: Polyvinylpyrrolidone
USP: United States pharmacopeia
UV: Ultraviolet
XRD: X-ray diffraction
