# Supplementary material for: The Combined Use of Imaging Approaches to Assess Drug Release from Multicomponent Solid Dispersions
Source: Pharm Res. 2016 Aug 29;34(5):990–1001. doi: 10.1007/s11095-016-2018-x (PMC5382183; doi:10.1007/s11095-016-2018-x)
Supplement: Supplementary file 1 — (DOCX 396 kb) [file 11095_2016_2018_MOESM1_ESM.docx]

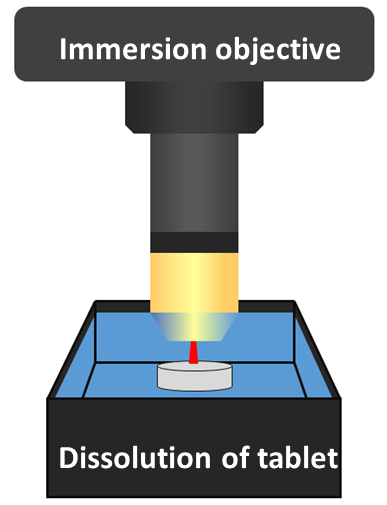
a)
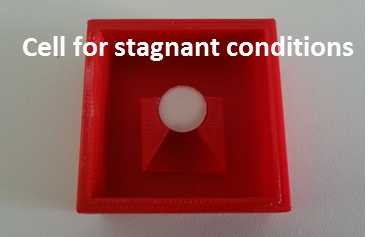


b)
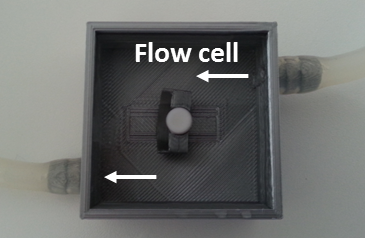
 c)

Supplementary Information 1: 3D printed cells enabling the measurement of tablet dissolution under water a) in stagnant conditions and b) in flow. c) Scheme of an immersion objective focused on the tablet surface.
