# Supplementary material for: The Combined Use of Imaging Approaches to Assess Drug Release from Multicomponent Solid Dispersions
Source: Pharm Res. 2016 Aug 29;34(5):990–1001. doi: 10.1007/s11095-016-2018-x (PMC5382183; doi:10.1007/s11095-016-2018-x)
Supplement: Supplementary file 2 — (DOCX 1120 kb) [file 11095_2016_2018_MOESM2_ESM.docx]

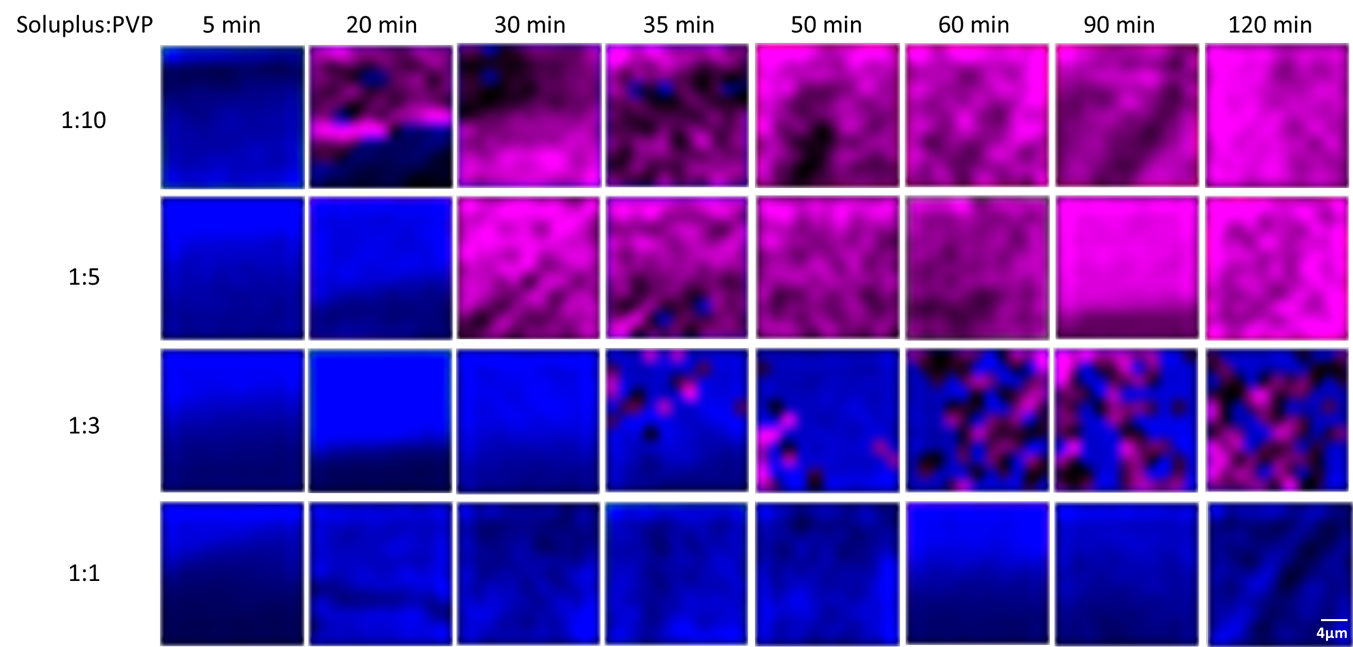


Supplementary Information 2: Raman images of a tablet surface at different times during dissolution showing the effect of admixed Soluplus on the inhibition of crystallization. False-colors indicate crystalline drug in pink and amorphous solid dispersion in blue. The dimensions of each image are 20 x 20 µm^2^.
